# Biosorption Performance of Encapsulated *Candida krusei* for the removal of Copper(II)

**DOI:** 10.1038/s41598-017-02350-7

**Published:** 2017-05-19

**Authors:** Chi Him Jim Luk, Joanne Yip, Chun Wah Marcus Yuen, Siu Kwong Pang, Kim Hung Lam, Chi Wai Kan

**Affiliations:** 1 Institute of Textiles and Clothing, The Hong Kong Polytechnic University, HungHom, Hong Kong; 2 Department of Applied Biology and Chemical Technology, The Hong Kong Polytechnic University, HungHom, Hong Kong

## Abstract

The use of microorganisms in biosorption is one of the most promising ways to remove trace amounts of heavy metal ions. Nevertheless, the enhancement of the successful removal of heavy metal ions by using different combinations of biosorbents is not generally guaranteed which leaves room to explore the application of the technique. In this study, the performance of free and immobilized forms of a yeast strain, *Candida krusei* (*C*. *krusei*), and calcium alginate (CaAlg) are evaluated for their ability to remove copper(II). Infrared spectroscopy, studies on the effects of pH and temperature, and kinetics and isotherm modelling are carried out to evaluate the biosorption. The infrared spectroscopy shows that the primary biosorption sites on the biosorbents are carboxylate groups. In addition, a higher pH and higher temperatures promote biosorption while a decline in biosorption ability is observed for *C*. *krusei* at 50 °C. The kinetics study shows that *C*. *krusei*, CaAlg and immobilized *C*. *krusei* (MCaAlg) conform with good correlation to pseudo-second order kinetics. MCaAlg and CaAlg fit well to the Langmuir isotherm while *C*. *krusei* fits well to the Temkin isotherm. From the experimental data, encapsulating *C*. *krusei* showed improved biosoprtion and address clogging in practical applications.

## Introduction

Many industries today use heavy metal ions; for example, the electroplating, metal mining and smelting industries^1^. The resultant discharged waste usually contains trace amounts of the corresponding metal ions in low parts per million (ppm) concentrations which still have detrimental effects to the environment as well as human and other living beings. Copper(II) is one of the most commonly used metals in different industries and one of the essential trace elements for mammals. However, excess intake of copper will mean adverse health effects, such as vomiting and gastrointestinal distress, and the chronic effects include damage to the liver and kidneys^2^. The World Health Organization (WHO) has provided a provisional guideline in which the upper levels of copper(II) intake should not exceed 2 mg L^−1^ ^3^. Conventional methods like precipitation, coagulation, ion exchange, membrane processing, and electrochemical approaches are effective in the rejection and recovery of metal ions but usually costly and some of the procedures are complicated^4–8^. For example, metal species can be recovered from target solutions through electrochemical approaches but the power supply would be expensive and complicated conditioning protocols are required for mixed metal solutions. Nanofiltration has been one such method that is being extensively studied recently. Although the technique works well to remove large amounts of pollutants that are not limited to heavy metal ions, the costs of the operation of nanofiltration and filter regeneration are concerns. On the other hand, biosorption has been studied as an alternative means of removing pollutants and demonstrated satisfactory removal ability over the past decades. Among the various types of biosorbents, microorganisms have been extensively explored due to their high removal efficiency, low cost, and simplicity in application^8–16^. However, the practical application of microorganisms in waste water treatment is problematic as the small particle size and low strength of microbial cells can cause the clogging of flow lines and filter parts, which usually occur during operation^16, 17^. To address these issues, the immobilization and encapsulation of microorganisms with a polymeric matrix have been subsequently developed and studied. Commonly adopted polymeric matrices include sodium alginate (Alg-Na), polyvinyl alcohol, polysulfone, chitosan, polyurethane, etc.^18–20^. Among the microorganisms studied for metal ion biosorption (bacteria, yeasts, fungi and algae)^21–27^, the *Candida* species (sp.) is one of more popular microbes that have been explored and show satisfactory biosorption performance^28–32^. In this study, *Candida krusei* (*C*. *krusei*), which has not been explored in depth for the removal of heavy metal ions, is experimentally investigated and encapsulated into calcium alginate (CaAlg) beads for the removal of copper(II) ions from an aqueous medium. Batch biosorption experiments are conducted and the removal mechanism is also evaluated.

## Materials and Methods

### Materials

Alg-Na (derived from *Laminaria hyperborean*) and calcium chloride (CaCl_2_, analytical grade) were obtained from UniChem Co. Ltd. Nitric acid (HNO_3_), potassium bromide (KBr, Fourier transform infrared (FTIR) grade) and copper standard for AAS (1000 mg L^−1^ copper in 2% HNO_3_) were obtained from Sigma-Aldrich LLC. Sodium hydroxide (NaOH) was obtained from VWR International LLC. All of the copper(II) solutions used in the batch experiments were derived after dilution of 1000 mg L^−1^ AAS copper standard solution with deionized water, and all pH adjustments were carried out by adding different concentrations of HNO_3_ and NaOH. *C*. *krusei* (ATCC 14243) was obtained from ATCC Co. The culture medium (yeast mold (YM) broth and agar were obtained from BD Co. The yeast pellet was rehydrated and inoculated in accordance with ATCC procedures^33^. Cultured *C*. *krusei* was harvested after an inoculation period of 24 hours at a temperature of 30 °C in 50 ml of broth medium in a 250 ml baffled-bottom flask.

### Preparation of calcium alginate beads

**Figure 1:**
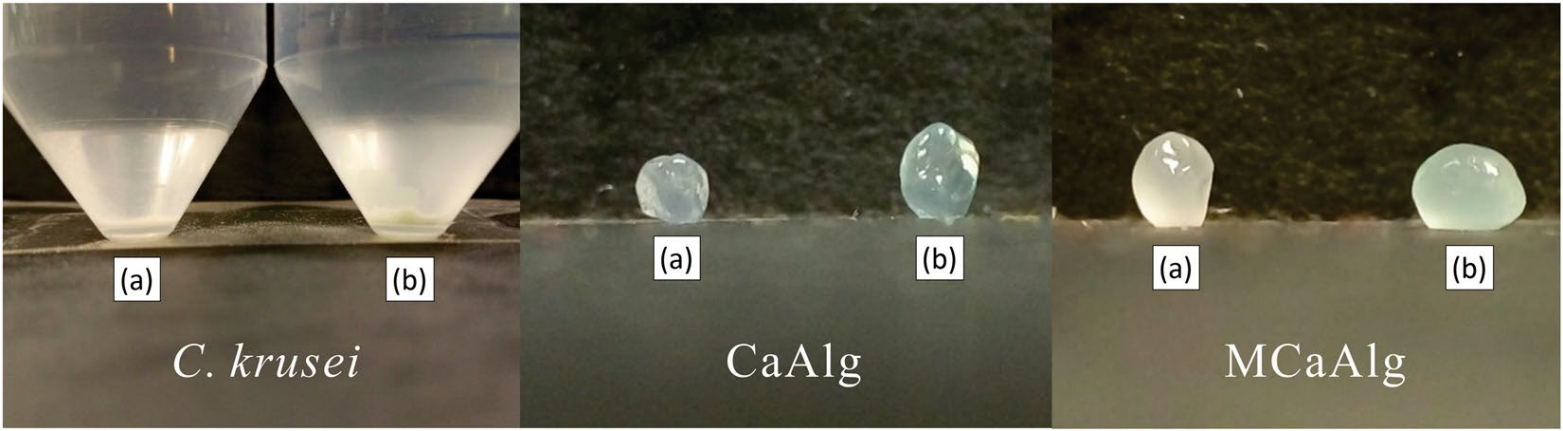
*C*. *krusei*, CaAlg and MCaAlg (**a**) before and (**b**) after copper(II) removal.

The Alg-Na solution (3.6% (w/v)) was prepared by dissolving Alg-Na in deionized water overnight. After complete dissolution was achieved, the solution was centrifuged at 6000 rpm for 10 minutes and the supernatant (from the Alg-Na solution) was gathered to produce the beads. The Alg-Na solution was diluted with deionized water at a volume ratio of 1:1 and then transferred into a syringe equipped with a 25 G needle tip. The solution was dripped into 50 mM CaCl_2_ solution. The ratio of the Alg-Na solution to the CaCl_2_ solution was 1:20 in volume. Beads (CaAlg) immediately formed after the solution was dripped into a CaCl_2_ hardening bath. The beads were post-hardened for 60 minutes. To prepare the *C*. *krusei*-encapsulated calcium alginate beads (MCaAlg), the prepared culture was mixed with the Alg-Na solution at a volume ratio of 1:1. The mixture was then dripped into the CaCl_2_ solution as mentioned above and subsequently hardened for 60 minutes. After the post-hardening period, both CaAlg and MCaAlg were filtered and rinsed with deionized water for subsequent batch biosorption experiments in which all of the beads were freshly prepared prior to the experiments. Figure 1(a,b) show the three biosorbents before and after the removal of copper(II). The average diameter of the CaAlg and MCaAlg beads is 2.1 mm and 2.5 mm, respectively.

### Fourier Transform Infrared Spectroscopy

The FTIR spectra of the CaAlg, MCaAlg and *C*. *krusei* were obtained by using a Perkin-Elmer Spectrum 100 FTIR spectrometer. Dried and pressed pellets were made by grinding different samples with FTIR grade KBr with an agate mortar.

### Batch Biosorption Experiments

The effects of pH and temperature on the biosorption of copper(II) with *C*. *krusei*, CaAlg and MCaAlg were examined. To determine the effects of the pH, copper(II) solutions (1.5 mM, 20 ml, 30 °C) at various pH values (1.2, 2.0, 3.1, 4.1, and 5.2) were prepared and adjusted by using HNO_3(aq)_ and NaOH_(aq)_. To determine the effects of temperature, copper(II) solutions (1.5 mM, 20 ml, pH 5.2) were prepared at temperatures of 30 °C, 40 °C and 50 °C. After the metal solutions were pre-heated to the desired temperatures, the biosorbents (2 ml CaAlg, 2 ml MCaAlg and 1 ml cultured *C*. *krusei*) were mixed with the solution and transferred to a shaking bath running at 155 rpm (orbital shaking bath). The initial concentrations of the copper(II) solution were the same in each batch. An aliquot of 100 μl was withdrawn and diluted accordingly for analysis at particular time intervals. Both the experiments on the effects of pH and temperature were conducted for 24 hours. The metal ion concentration was determined by using a Perkin Elmer AAnalyst 800 atomic absorption spectrometer at 592 nm. The amount of adsorbed copper(II) at equilibrium (*q*_*e*_, mmol g^−1^) was determined with Equation (1):

1 $${q}_{e}=\frac{({C}_{0}-{C}_{e})\times V}{m}$$

where *C*_*0*_ is the initial concentration of the copper(II) solution in mM and *C*_*e*_ is the concentration of the copper(II) solution at the equilibrium state; m is the mass of the biosorbent and *V* (L) is the volume of the metal solution.

Kinetics experiments were performed by transferring the biosorbents (2 ml CaAlg, 2 ml MCaAlg and 1 ml cultured *C*. *krusei*) into 20 ml of 0.05 mM copper(II) solution at a temperature of 30 °C and pH of 5.2 for 24 hours. The initial concentrations of the copper(II) solution were the same in each batch. The adsorbed amount of copper(II) at time t (*q*_*t*_, mmol g^−1^) was determined by using Equation (2):

2 $${q}_{t}=\frac{({C}_{0}-{C}_{t})\times V}{m}$$

where *C*_*0*_ (mM) is the initial concentration of the copper(II) solution, *C*_*t*_ (mM) is the concentration at a pre-determined time interval, m is the mass of the biosorbent and *V* (L) is the volume of the metal solution.

Isotherm studies were conducted with 20 ml of copper(II) solution in a range of concentrations (from 0.05 mM to 1.5 mM) at 30 °C and pH of 5.2. CaAlg (2 ml), MCaAlg (2 ml) and cultured *C*. *krusei* (1 ml) were incorporated into the metal solution and the equilibrium metal concentrations *C*_*e*_ were measured after 24 hours of biosorption. Langmuir, Freundlich and Temkin isotherm equations were used to evaluate the sorption equilibrium. The Langmuir isotherm assumes that there is monolayer adsorption onto a homogeneous surface. All of the adsorption sites are equivalent and no interactions occur between the adsorbed adsorbates on the surface with the use of isotherm calculations^32^. The Freundlich isotherm applies to adsorption onto a heterogeneous surface and not limited to low adsorbate concentrations^32^. The Temkin isotherm assumes that the heat of adsorption of all the molecules in a layer decreases linearly with coverage due to adsorbate-adsorbent interactions^34^. The linearized mathematical expressions of the Langmuir (Equation (3)), Freundlich (Equation (4)) and Temkin (Equations (5) and (6)) isotherms are shown below:

3 $$\frac{1}{{q}_{e}}=\frac{1}{{q}_{max}}+\frac{1}{{q}_{max}{K}_{L}}\frac{1}{{C}_{e}}$$

4 $$\mathrm{ln}\,{q}_{e}=\,\mathrm{ln}\,{K}_{F}+\frac{1}{n}\,\mathrm{ln}\,Ce$$

5 $${q}_{e}=B\,\mathrm{ln}\,{A}_{T}+B\,\mathrm{ln}\,Ce$$

6 $$B=\frac{RT}{{b}_{T}}$$

where *q*_*e*_ (mmol g^−1^) is the amount of copper(II) adsorbed by the biosorbents at equilibrium; *q*_*max*_ (mmol g^−1^) and *K*_*L*_ (L mmol^−1^) are the Langmuir isotherm constants which denote the maximum adsorption capacity of the biosorbents of copper(II) at equilibrium and the intensity of the adsorption respectively; *n* and *K*_*F*_ (L g^−1^) are the Freundlich isotherm constants in which *K*_*F*_ is the distribution coefficient; *B*, *b*_*T*_ (J mmol ^−1^) and *A*_*T*_ (L g^−1^) are the Temkin isotherm constants, in which *B* is a constant that is related to the heat of adsorption and *A*_*T*_ is an equilibrium binding constant; *C*_*e*_ (mM) is the concentration of the copper(II) solution at equilibrium; *R* is a universal gas constant and *T* is the temperature in a particular condition.

## Results and Discussion

### Infrared spectroscopy analysis

**Figure 2:**
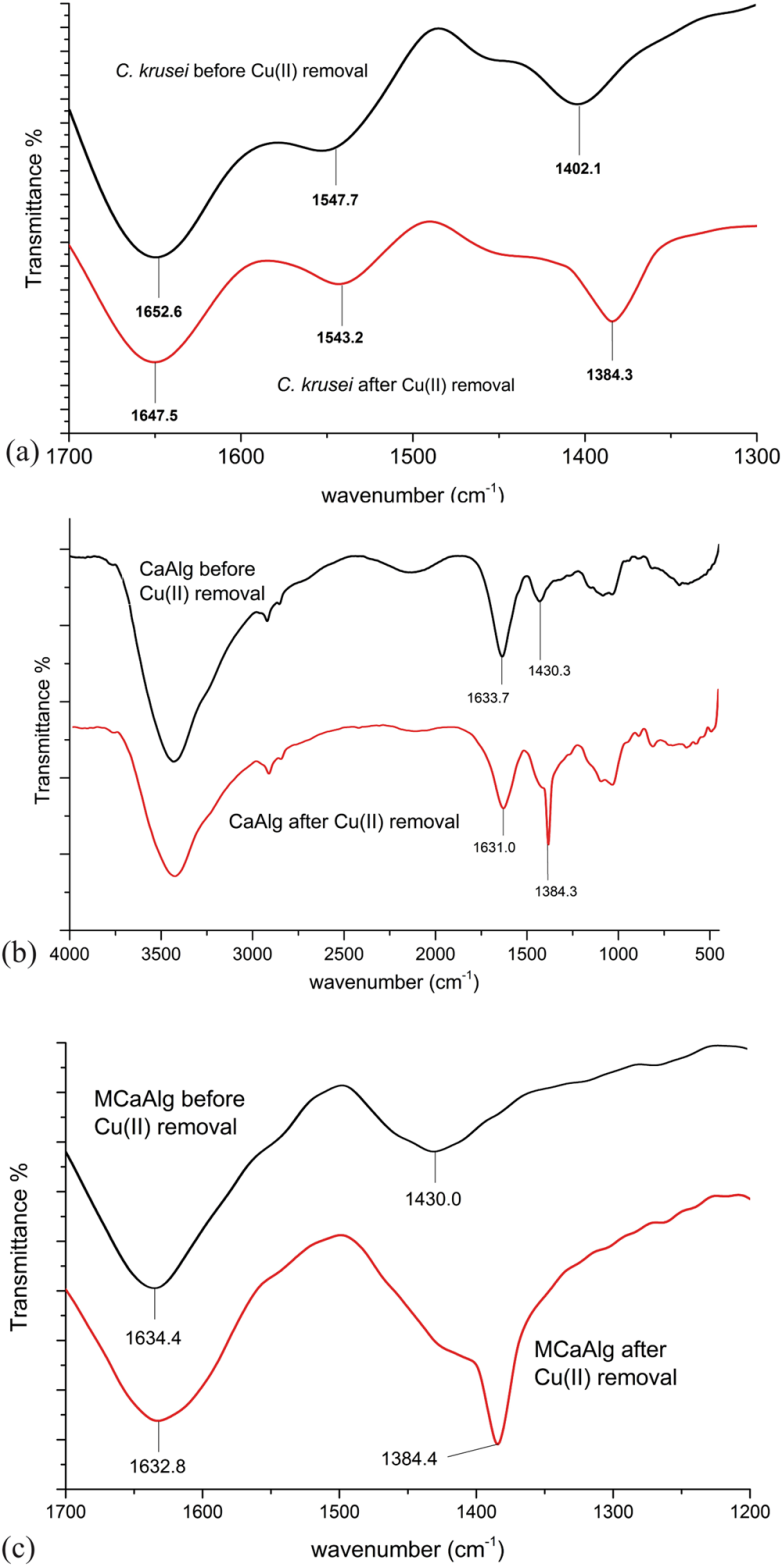
IR spectrum of (**a**) *C*. *krusei* (**b**) CaAlg (**c**) MCaAlg before and after copper(II) removal.

Figure 2(**a–c)** show the region of an infrared (IR) spectrum that is between 1200 cm^−1^ to 1700 cm^−1^ for *C*. *krusei*, CaAlg and MCaAlg before and after the biosorption of copper(II) respectively. The polymeric network of CaAlg is composed of covalently linked consecutive pairs of alginic acid salts^34,35]^, with calcium ions as the divalent cross-linking agent. Alginic acid is structurally similar to D-mannose and its C-6 position is a carboxylate group instead of a hydroxyl group. Since both CaAlg and MCaAlg are mainly composed of CaAlg polymeric networks, they share a similar IR spectrum. On the other hand, *C*. *krusei* is a yeast species with a cell wall that is mainly composed of chitin (poly-*N*-acetylglucosamine), glucan (poly-D-glucose) and different proteins^36^. Hence, the IR spectrum of *C*. *krusei* shows the presence of amino N-H (1543–1647 cm^−1^) and carboxylate COO^−^ (1384–1402 cm^−1^) absorption peaks. After the biosorption of copper(II), the absorption band shifted and a new absorption band was observed with CaAlg and MCaAlg. The carboxylate COO^−^ of *C*. *krusei* shifted from 1402.1 cm^−1^ to 1384.3 cm^−1^; new asymmetric and symmetric COO^−^ absorption bands of CaAlg and MCaAlg appeared at approximately 1384.3 cm^−1^ respectively. Before the biosorption of copper(II), the alginate polymeric network of CaAlg and MCaAlg was bridged in the presence of the calcium divalent cations and the asymmetric and symmetric COO^−^ absorbed the IR at around 1430 cm^−1^. After the biosorption of copper(II), an absorption peak at 1384 cm^−1^ appeared. This means that the counter ions in the polymeric matrix changed, and most likely due to the presence of the copper(II) ions. On the other hand, the carboxylate COO^−^ absorption band of *C*. *krusei* shifted from 1402.1 cm^−1^ to 1384.3 cm^−1^, which means that the major biosorption site for copper(II) removal is the carboxylate COO^−^ group on the cell wall.

### Effects of pH on copper(II) removal

**Figure 3:**
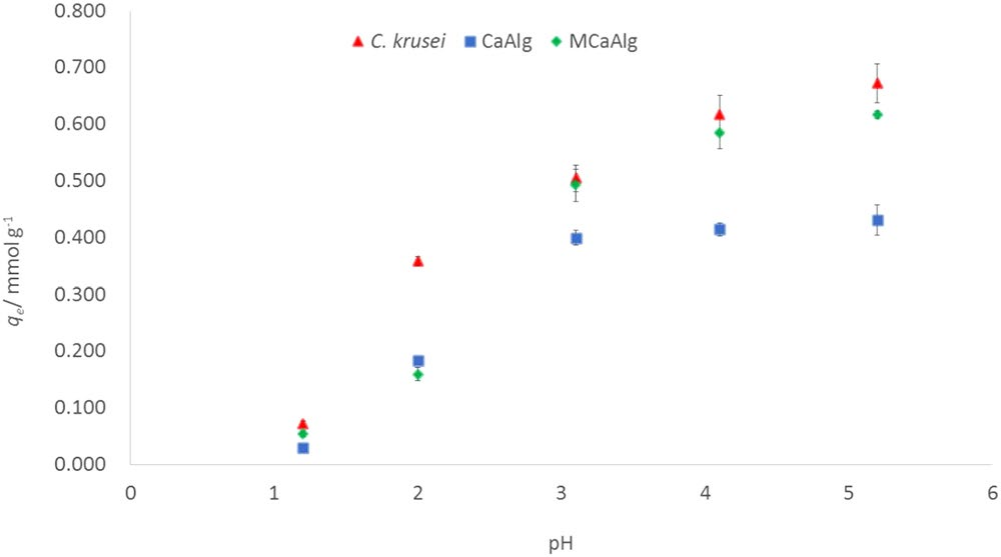
Effects of pH on removal of copper(II) with *C*. *krusei*, CaAlg and MCaAlg.

Figure 3 shows the effects of pH on the removal of copper(II) ions by using *C*. *krusei*, CaAlg and MCaAlg with a pH of 1.2 to 5.2. pH values beyond 5.2 were not examined since they might give rise to the precipitation of copper(II) metal species. A higher removal efficiency was observed by increasing the pH of the bulk solution. The optimal pH for copper removal by using *C*. *krusei* (0.687 mmol g^−1^), CaAlg (0.431 mmol g^−1^) and MCaAlg (0.617 mmol g^−1^) is 5.2. In examining the metal ion removal process, the speciation of the metal ions at different pHs and the interactions between the biosorbent and metal ions were taken into consideration. At acidic pHs, copper(II) predominantly exists as the Cu^2+^ form. When the removal process was subjected to very low pH environments (pH 1.2 to 2), the carboxylate COO^−^ found on the biosorbents was largely protonated i.e. R-COOH. The coordination of copper(II) ions to the protonated carboxylate groups is less likely to occur, and hence resulted in very little removal. When the environment was less acidic, the amount of the conjugated form of the carboxylate COO^−^ on the biosorbents increased. At an optimal pH of 5.2, the interaction between the biosorbents and copper(II) ions was optimized. This is related to the FTIR analysis in which the coordination of copper(II) is the primary removal mechanism between copper(II) and the biosorbents. From the experiment on the effects of pH, it was observed that at a pH of 5.2, *C*. *krusei* has the highest *q*_*e*_, followed by MCaAlg with the least amount by CaAlg. Although *C*. *krusei* alone has the highest *q*_*e*_ among the three types of biosorbents, there are separation problems in practical applications. By encapsulating *C*. *krusei*, the *q*_*e*_ is slightly reduced compared to the use of only *C*. *krusei*. However, the performance of encapsulated *C*. *krusei* (MCaAlg) still excels that of CaAlg. This is because the encapsulation technique compensates for the shortcomings of only using *C*. *krusei* or CaAlg by itself.

### Effects of temperature on copper(II) removal

**Figure 4:**
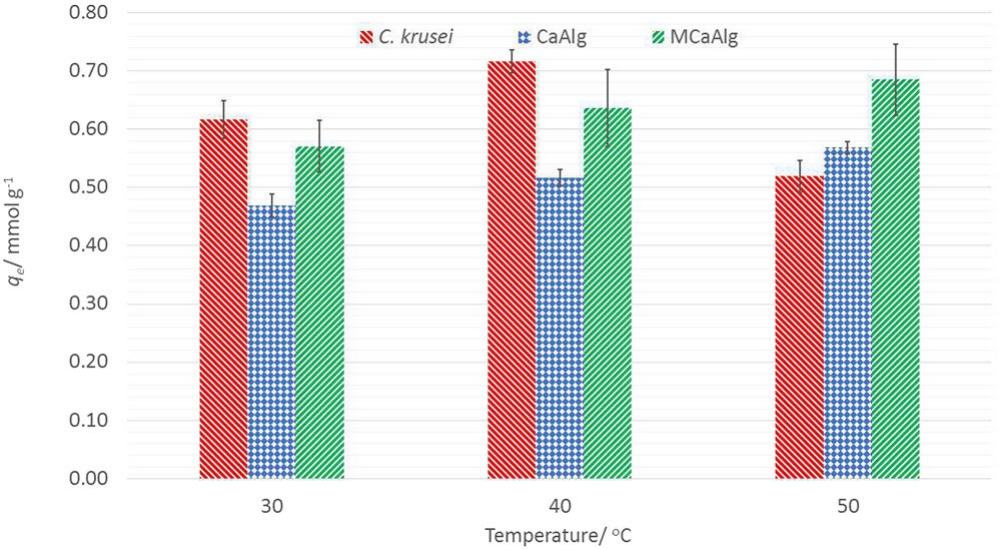
Effects of temperature on removal of copper(II) with *C*. *krusei*, CaAlg and MCaAlg.

Figure 4 shows the effects of temperature on the removal of copper(II) with the use of the three biosorbents at temperatures of 30 °C, 40 °C and 50 °C. It can be observed that there is an increase in the ability of *C*. *krusei* to remove copper(II) when the temperature is increased from 30 °C (0.616 mmol g^−1^) to 40 °C (0.716 mmol g^−1^) but when the temperature is further increased to 50 °C, this ability is reduced (0.419 mmol g^−1^). On the other hand, for both CaAlg and MCaAlg, it can be observed that there is a trend of increase with temperature increases; that is, 0.469 mmol g^−1^ at 30 °C, 0.517 mmol g^−1^ at 40 °C and 0.568 mmol g^−1^ at 50 °C with the use of MCaAlg and 0.571 mmol g^−1^ at 30 °C, 0.637 mmol g^−1^ at 40 °C and 0.685 mmol g^−1^ at 50 °C with the use of MCaAlg. Aside from a general trend of increase in ability to remove copper(II), MCaAlg also demonstrated a higher capacity for the removal of copper(II) at all three temperatures in comparison to CaAlg. Therefore, the results showed that with increasing temperature, CaAlg and MCaAlg both demonstrate better capacity to remove copper(II) while this is not the case with the use of *C*. *krusei*. This is because in CaAlg and MCaAlg, the coordination of copper(II) to the carboxylate groups was facilitated by increased temperature. Even though this is the case, biosorption with the use of *C*. *krusei* still resulted in increased copper(II) removal when the temperature was increased from 30 °C to 40 °C because a similar biosorption mechanism is used. A possible reason for the increase in biosorption could be attributed to an environment that is conducive for the biosorption of copper(II) at 40 °C. However, further increases in the temperature to 50 °C would probably give rise to the denaturation of the enzymes which changes the good biosorption ability of *C*. *krusei*, and reduces the uptake of copper(II)^37^. The effect of temperature on the biosorption ability of *C*. *krusei* implies that the process of absorption is not solely physical, but biological processes are also involved. We observed that MCaAlg has the highest *q*_*e*_ at high temperatures while the opposite was true for *C*. *krusei* which had the lowest *q*_*e*_, possibly because the enhancement in biosorption ability by using CaAlg dominates the suppression from the denaturation of *C*. *krusei*. In addition to the results obtained from examining the effect of pH, MCaAlg also showed improved performance when the biosorption temperature was no longer optimal when naked *C*. *krusei* was used. Therefore, encapsulation can improve biosorption when the bulk environment is not appropriate for naked *C*. *krusei* to remove the targeted biosorbates.

### Kinetics studies on copper(II) removal

Kinetics studies are one of the important ways to evaluate the removal efficiency of adsorbents, removal mechanisms of sorption systems and rate constants. Several kinetics equations to do so have been developed which include, but not limited to, the pseudo-first order^38^, pseudo-second order^39^, and intraparticle diffusion models^40^. A summary is provided in Table 1 of the linear mathematical expressions of the three kinetics models.

**Table 1:** Linear forms of pseudo-first order, pseudo-second order and intraparticle diffusion models.

Kinetic model	Linear form
Pseudo-first order model	$\mathrm{log}({q}_{e}-{q}_{t})=\,\mathrm{log}\,{q}_{e}-\frac{{k}_{1}}{2.303}t$
Pseudo-second order model	$\frac{t}{{q}_{t}}=\frac{1}{{k}_{2}{q}_{e}^{2}}+\frac{1}{{q}_{e}}t$
Intraparticle diffusion model	${q}_{t}={k}_{i}\cdot {t}^{0.5}$

*k*_*1*_, *k*_*2*_, and *k*_*i*_ are the rate constant of corresponding kinetics model; *q*_*e*_ is the amount of adsorbed copper(II) at equilibrium and *q*_*t*_ is the amount of copper(II) adsorbed at time *t* (mmol g^−1^).

**Figure 5:**
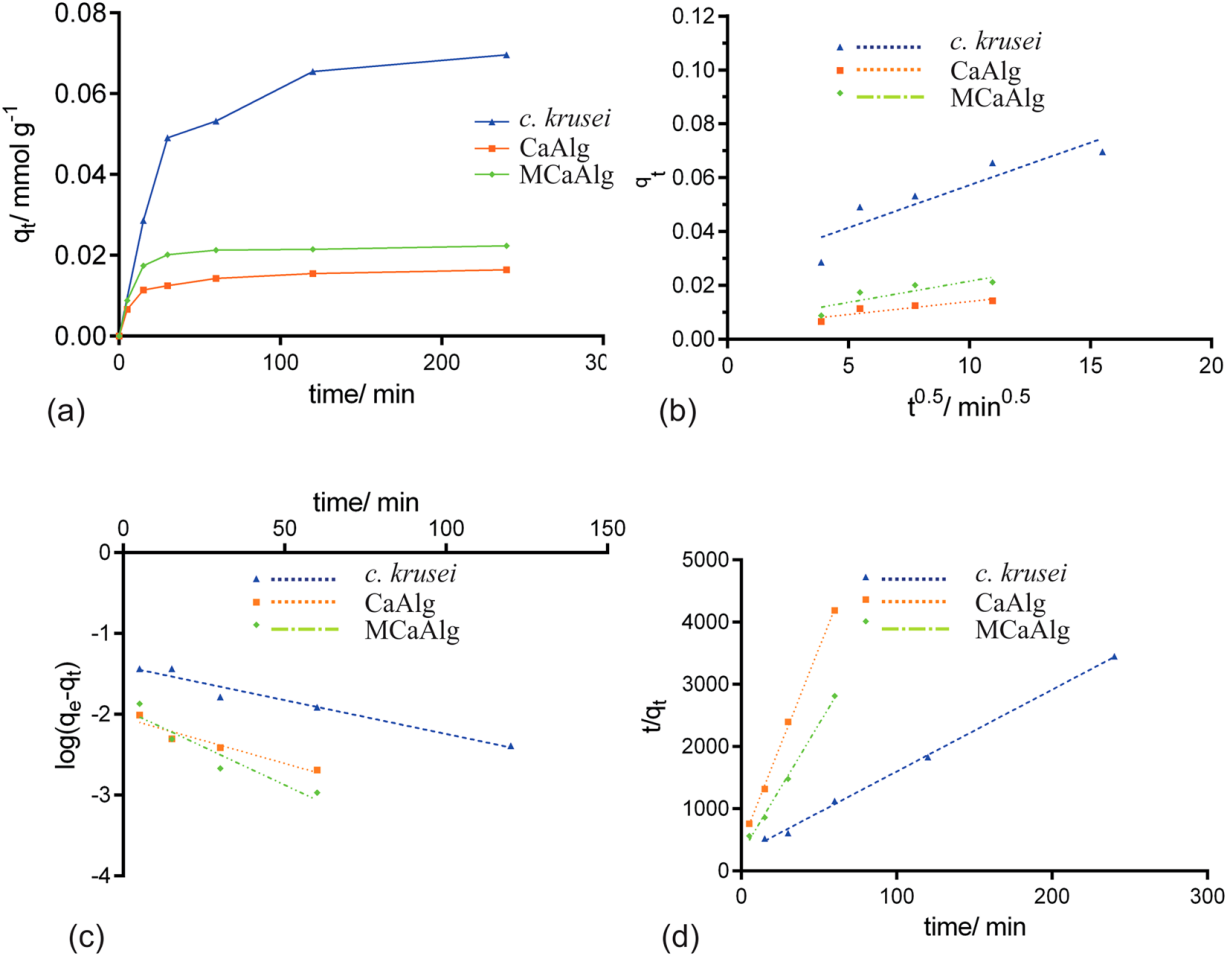
Kinetics studies on copper(II) removal (**a**) with *C*. *krusei*, CaAlg and MCaAlg (**b**) Intraparticle diffusion modelling (**c**) Pseudo-first-order modelling (**d**) Pseudo-second-order modelling.

Figure 5a–d show copper(II) removal with the use of *C*. *krusei*, CaAlg and MCaAlg through intraparticle diffusion, and pseudo-first order and pseudo-second order modelling respectively at 30 °C and pH 5.2 with 0.05 mM of copper(II). Table 2 summarizes the kinetics parameters of the modelling.

**Table 2:** Kinetics parameters of *C*. *krusei*, CaAlg and MCaAlg upon removal of copper(II).

	Intraparticle diffusion					
	*C*. *krusei*		CaAlg		MCaAlg	
***k***_***i***_ (mmol g^−1^ min^−0.5^)	0.0031		0.0007		0.0009	
R^2^	0.8217		0.8432		0.6457	
	***q***_***e***_ ** mmol g** ^**−1**^	**Pseudo-first order**			**Pseudo-second order**	
		***k***_***1***_ (**min** ^**−1**^)	**R** ^**2**^	***q***_***e***_	***k***_***2***_ (**g mmol** ^**−1**^ **min** ^**−1**^)	**R** ^**2**^
*C*. *krusei*	0.0384	0.0190	0.9431	0.0760	0.6031	0.9982
CaAlg	0.0091	0.0259	0.9248	0.0159	9.1000	0.9986
MCaAlg	0.0116	0.0431	0.8950	0.0241	5.9994	0.9962

From the plots, it can be observed that CaAlg and MCaAlg reach equilibrium at 60 minutes after contact while *C*. *krusei* reach equilibrium at 240 minutes. Among the three kinetics models, all three biosorbents show the highest degree of correlation to the pseudo-second order kinetics (R^2^ of *C*. *krusei* = 0.9982; R^2^ of CaAlg = 0.9986; R^2^ of MCaAlg = 0.9962) compared to the pseudo-first order kinetics (R^2^ of *C*. *krusei* = 0.9431; R^2^ of CaAlg = 0.9248; R^2^ of MCaAlg = 0.8950). The pseudo-second order kinetics therefore best describe the three types of biosorptions. This is in agreement with the research work in Hassan *et al*.^41^ which focused on the ionotropic cross-linking of metal alginate. They showed that the cross-linking of alginates with divalent metal ions at a stoichiometric ratio of 2:1 which correlates with this result of the pseudo-second-order kinetics fitting. On the other hand, the intraparticle diffusion model describes diffusion-controlled kinetics in that any fitting process would pass through the origin when plotted and fitted. In Fig. 5(b), all three plots of the biosorption do not pass through the origin. The biosorptions are therefore most likely not diffusion-controlled kinetics but rather chemical processes.

### Isotherm modelling on copper(II) removal

**Figure 6:**
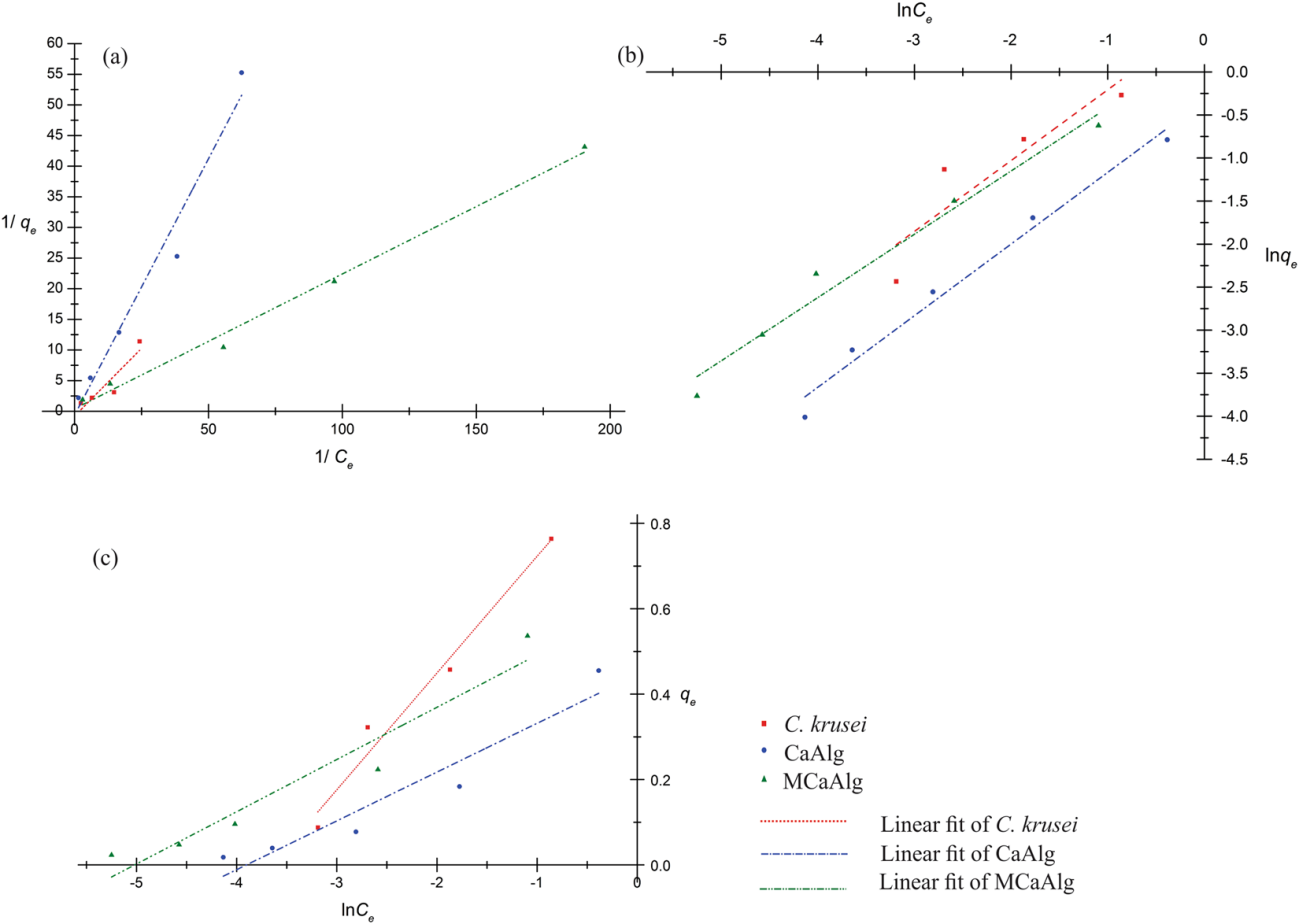
Isotherm modelling on copper(II) removal (**a**) *C*. *krusei*, CaAlg and MCaAlg fit in Langmuir isotherm (**b**) *C*. *krusei*, CaAlg and MCaAlg fit in Freundlich isotherm. (**c**) *C*. *krusei*, CaAlg and MCaAlg fit in Temkin isotherm.

Figure 6a–c show the fit of the three biosorbents in the Langmuir, Freundlich and Temkin isotherms for the removal of 0.05 mM copper(II) at 30 °C and pH 5.2. Table 3 shows the corresponding isotherm constants. *C*. *krusei* (R^2^ = 0.9625) fits well to the Temkin isotherm. Both MCaAlg and CaAlg are fitted in the Langmuir and Freundlich isotherms with a correlation coefficient >0.96, where the former is fitted with a *q*_*max*_ of 2.418 mmol g^−1^. The Langmuir isotherm assumes homogeneous and monolayer adsorption while the Freundlich isotherm assumes heterogeneous adsorption. Although both isotherm models are a good fit, the Langmuir isotherm is more applicable because from previous results and discussions, the biosorptions are shown to be mainly as the coordination of copper(II) ions to carboxylate COO^−^ groups on CaAlg and MCaAlg and these carboxylate groups have the same chemical properties and heat of adsorption. On the other hand, *C*. *krusei*, which coordinates copper(II) ions to the carboxylate groups, has a more complex surface structure compared to CaAlg and MCaAlg. Therefore, *C*. *krusei* is not well fitted to both the Langmuir and Freundlich isotherms but only to the Temkin isotherm. This result is consistent with the study on the effect of temperature in that free *C*. *krusei* behaves differently in the encapsulated form. The Langmuir isotherm fitting of CaAlg, however, showed a negative intercept, hence a negative *q*_*max*_ and *K*_*L*_ were interpreted. Nevertheless, the error is statistical so that the isotherm fitting should be determined by including the previous results and discussions. Therefore, instead of the Freundlich isotherm, the Langmuir isotherm was used to study the biosorption of CaAlg. On the other hand, MCaAlg is well fitted to the Langmuir isotherm and this correlates with the reported results. The good fit of the Temkin isotherm with *C*. *krusei* indicates that the biosorption adheres to the assumption that the heat of adsorption is reduced upon coverage due to adsorbate-adsorbent interactions.

**Table 3:** Isotherm constants of *C*. *krusei*, CaAlg and MCaAlg on removal of copper(II).

	*C*. *krusei*	CaAlg	MCaAlg
Langmuir constants			
*q*_max_ (mmol g^−1^)	−1.267	−0.645	2.418
*K*_*L*_ (L mmol^−1^)	−1.785	−1.550	1.881
R^2^	0.76	0.96	0.99
Freundlich constants			
*n*	1.216	1.2017	1.3959
*K*_*F*_ (L g^−1^)	1.851	0.7155	1.2588
R^2^	0.74	0.98	0.97
Temkin constants			
*B* (J mmol^−1^)	0.273	0.114	0.122
*b*_*T*_	9.213	22.05	20.60
*A*_*T*_ (L g^−1^)	38.23	49.59	151.3
R^2^	0.96	0.89	0.91

## Conclusions

The use of microorganisms in biosorption is one of the most promising ways to remove trace amounts of heavy metal ions. However, practical applications are problematic due to clogging issues. In this study, *C*. *krusei* is experimentally investigated and encapsulated into CaAlg beads for the removal of copper(II) ions from an aqueous medium.

An examination of the effect of pH reveals that a less acidic environment leads to a higher biosorption capacity of all three biosorbents. The effects of temperatures of 30 °C, 40 °C and 50 °C are examined, which show that CaAlg and MCaAlg have better biosorption capacity with increasing temperature while the optimum temperature for the biosorption of *C*. *krusei* is 40 °C. Infrared spectroscopy, kinetics studies and isotherm modelling are used to evaluate the removal mechanism of copper(II). According to the infrared spectroscopy results, the biosorptions are mainly carried out with the chelation of copper(II) to carboxylate COO^−^ on the three biosorbents. The biosorption kinetics of the three biosorbents follow pseudo-second order instead of pseudo-first order or the intraparticle diffusion model. From the isotherm modelling, it is found that the Langmuir isotherm best represents the biosorption equilibrium of MCaAlg and CaAlg while *C*. *krusei* fits well to the Temkin isotherm. Overall, although the biosorption capacity of MCaAlg falls short of *C*. *krusei*, encapsulation would address the concerned separation problems caused by clogging which is resultant of using only microbes. Furthermore, compared to the use of only polymer, encapsulation also enhances biosorption. The encapsulation of microbes has therefore demonstrated their good ability to remove metal ions in water treatment and remedy separation processes.
